# Longitudinal Study for the Detection and Quantification of *Campylobacter* spp. in Dairy Cows during Milking and in the Dairy Farm Environment

**DOI:** 10.3390/foods12081639

**Published:** 2023-04-13

**Authors:** Anna-Delia Knipper, Steven Göhlich, Kerstin Stingl, Narges Ghoreishi, Carola Fischer-Tenhagen, Niels Bandick, Bernd-Alois Tenhagen, Tasja Crease

**Affiliations:** 1 Department of Biological Safety, German Federal Institute for Risk Assessment (BfR), Max-Dohrn-Straße 8-10, 10589 Berlin, Germany; steven.goehlich@bfr.bund.de (S.G.); kerstin.stingl@bfr.bund.de (K.S.); niels.bandick@bfr.bund.de (N.B.); bernd-alois.tenhagen@bfr.bund.de (B.-A.T.); tasja.crease@bfr.bund.de (T.C.); 2 Department Exposure, German Federal Institute for Risk Assessment (BfR), Max-Dohrn-Straße 8-10, 10589 Berlin, Germany; narges.ghoreishi@bfr.bund.de; 3 Center for Protection of Experimental Animals, German Federal Institute for Risk Assessment (BfR), Max-Dohrn-Straße 8-10, 10589 Berlin, Germany; carola.fischer-tenhagen@bfr.bund.de

**Keywords:** food safety, food hygiene, raw milk, cattle, risk assessment

## Abstract

Campylobacteriosis outbreaks have repeatedly been associated with the consumption of raw milk. This study aimed to explore the variation in the prevalence and concentration of *Campylobacter* spp. in cows’ milk and feces, the farm environment and on the teat skin over an entire year at a small German dairy farm. Bi-weekly samples were collected from the environment (boot socks), teats, raw milk, milk filters, milking clusters and feces collected from the recta of dairy cows. Samples were analyzed for *Campylobacter* spp., *E. coli*, the total aerobic plate count and for *Pseudomonas* spp. The prevalence of *Campylobacter* spp. was found to be the highest in feces (77.1%), completely absent in milking equipment and low in raw milk (0.4%). The mean concentration of *Campylobacter* spp. was 2.43 log_10_ colony-forming units (CFU)/g in feces and 1.26 log_10_ CFU/teat swab. Only a single milk filter at the end of the milk pipeline and one individual cow’s raw milk sample were positive on the same day, with a concentration of 2.74 log_10_ CFU/filter and 2.37 log_10_ CFU/mL for the raw milk. On the same day, nine teat swab samples tested positive for *Campylobacter* spp. This study highlights the persistence of *Campylobacter* spp. for at least one year in the intestine of individual cows and within the general farm environment and demonstrates that fecal cross-contamination of the teats can occur even when the contamination of raw milk is a rare event.

## 1. Introduction

Campylobacteriosis, caused by bacteria of the genus *Campylobacter*, is the most commonly reported bacterial foodborne gastrointestinal infection in humans in the European Union (EU) [1]. Aside from *Salmonella* spp. and the shigatoxin-producing *Escherichia coli* (STEC), *Campylobacter* spp., predominantly *Campylobacter* (*C.*) *jejuni*, have been regarded as the most notable health hazards, with clear links between drinking raw milk and human illness [2,3,4]. Between 2011 and 2020, raw milk was one of the main food vehicles causing “strong evidence” for foodborne campylobacteriosis outbreaks reported in the EU [5].

Thermophilic *Campylobacter* spp. colonize the intestinal tract of cattle and are shed intermittently with the feces [6,7,8,9]. Therefore, it is assumed that in raw milk, this pathogen mainly originates from fecal cross-contamination during milking. However, it is not clear how this contamination takes place and how often raw milk is contaminated during milking [6,10,11,12].

The limited number of studies on and low concentrations of *Campylobacter* spp. along the raw milk supply chain have challenged previous risk assessments for raw milk consumption. Some studies investigated the prevalence of *Campylobacter* spp. in the bulk milk tank and milk filter. A meta-analysis of results from North America, Europe and New Zealand provided an estimated mean prevalence of 1.54% for *Campylobacter* spp. in bulk tank milk and 1.75% in milk filters [13]. Two studies attempted to quantify the contamination using the most probable number (MPN) method. They found 16 ± 30 MPN/100 mL in the bulk tank milk [14] and 1 MPN/21 mL in raw milk from farm vats [15]. Despite the low bacterial prevalence and concentration, the consumption of raw milk is considered a high-risk behavior [16]. Consumers are advised to boil raw milk prior to consumption to inactivate pathogens [2]. However, surveys in Italy found that 13.9% to 43% of consumers did not boil raw milk before consumption [17].

The prevalence of *Campylobacter* spp. in the feces of dairy cows vary widely from 0 to 100% [18]. The studies included in the meta-analysis differed in their design and the size of the herds investigated [18]. Seven studies reported quantitative data for *Campylobacter* spp. in cow feces [19,20,21,22,23,24,25]. The concentration ranged from 2 log_10_ colony-forming units (CFU)/g feces to 4 log_10_ CFU/g feces [20,21]. One study in New Zealand investigated the differences in the fecal concentrations of *C. jejuni* between individual cows based on rectal sampling [23]. Three cows on a pasture and three cows in confinement housing were grouped together as a “high-shedder group”, harboring a median concentration of 3–3.6 log_10_ *C. jejuni* per g of fresh feces [23].

Few studies have focused on the raw milk supply chain and the herd-level epidemiology of *Campylobacter* spp. [6,26,27,28,29]. To estimate the transmission of *Campylobacter* spp. from feces to milk, it is necessary to investigate both in the same setting. Limited longitudinal data on cross-contamination with *Campylobacter* spp. from feces to raw milk are available. Frequent sampling is required to detect contamination events and to estimate their frequency because the contamination of milk with *Campylobacter* spp. is expected to occur only sporadically. To the best of our knowledge, only one study from Norway provides concurrent qualitative prevalence data on *Campylobacter* spp. in cows’ feces, on teat skin, in raw milk and in environmental samples [30]. However, *Campylobacter* spp. were not quantified in that study.

In our study, the prevalence and concentration of *Campylobacter* spp. in fecal samples, teat swabs, raw milk, milk filters and boot sock samples from the stable alley were determined to close the gaps in the described knowledge and data. We determined the frequency of fecal shedding of *Campylobacter* spp. in individual cows and in different seasons. We compared the occurrence of *Campylobacter* spp. on teat skin, in raw milk and in milk filters with the fecal shedding of this pathogen. *Escherichia coli*, *Pseudomonas* spp. and the total aerobic colony count (TACC) were analyzed as indicators of fecal and environmental contamination throughout the milking process.

## 2. Materials and Methods

### 2.1. Sampling Site

A Holstein cow herd with 22 lactating animals in Berlin, Germany, was sampled over a period of one year, from the 19th of April 2021 to the 8th of April 2022. Cows were kept in a free-stall barn with a concrete floor and access to an outdoor sand paddock throughout the year. Samples were taken on two consecutive days every two weeks from primiparous (*n* = 19) and second lactation (*n* = 3; cow ID 4301, 4317, and 4320) cows. The animals were fed a diet consisting of 27.7% grass silage, 29.5% maize silage, 6.0% straw, 30.1% hay, 6.0% beet pulp, and 0.61% minerals ad libitum throughout the year. Concentrates were provided individually in a transponder-controlled automatic feeder to meet the energy requirements for 25 kg/d of energy-corrected milk. On the first day, samples were collected from raw milk, milk filters, teat skin and milking clusters at 6 a.m. Teat swabs and raw milk samples were taken from twelve lactating cows over the entire period. The same cows were sampled on every occasion with some exceptions: (1) dry cows on specific sampling days were replaced by lactating cows; (2) two cows had to be treated with antibiotics against mastitis during the trial period. These were excluded once from teat swab and raw milk sampling during treatment. These exceptions resulted in teat swab and raw milk samples from 21 different cows.

On the second day, rectal fecal samples were taken from twelve cows and boot sock samples were obtained from the stable corridor. A mathematical randomization fixed the twelve cows always used for rectal fecal sampling. One cow (6057) left the herd for a reason unrelated to the study and was replaced by a new cow (6005), who was used for rectal fecal sampling. Samples were transported on ice to the laboratory, and microbiological processing was carried out within two hours of sampling at the latest. The entire experiment was approved by the State Office for Health and Social Affairs (LAGESO) (Reg.-No.: G 0215/20).

### 2.2. Sample Preparation

#### 2.2.1. Teat Swab Samples

Two gauze pads (10 × 10 cm, 8-fold) were placed in a plastic bag and moistened with 8 mL 0.9% NaCl. The bag was closed and stored at 4 °C until sampling. One bag was used per cow. On two occasions, teat swabs could only be collected from 11 cows, resulting in a total of 286 teat swab samples (instead of 288, 24 × 12 cows) during the study.

All four teats of a cow were wiped with the two gauze pads while wearing gloves before being cleaned by the milkers with moist cleaning wipes (udder wipes, clean paper^®^, Lauchhammer, Germany). The gloves were changed between sampling each individual cow to avoid cross-contamination. In the laboratory, the gauze pads were visually scored to assess the fecal contamination of the teat skin. Four scoring categories were used: K1: gauze pad clean; K2: gauze pad colored yellowish; K3: gauze pad discolored brown, possibly with fecal particles; K4: gauze pad brownish in color, feces clearly visible on the gauze pads.

Twenty-five milliliters of 1% phosphate-buffered peptone water (PW) was added to each teat swab sample (consisting of two gauze pads) and homogenized using a GRINDOMIX GM200 (Retsch GmbH, Haan, Germany) for 120 s at 4000 rpm.

#### 2.2.2. Raw Milk Samples

While wearing gloves and after the teats were cleaned with udder wipes (clean paper^®^, Lauchhammer, Germany), raw milk samples were obtained from all four teats and pooled in 50 mL falcon tubes. No disinfectant was applied before the raw milk was sampled. On two occasions, raw milk samples could only be collected from 11 cows, resulting in a total of 286 raw milk samples (instead of 288, 24 × 12 cows) during the study.

#### 2.2.3. Milking Clusters Samples

After the completion of the milking process, one pooled sample was taken from each milking cluster (with four teat cups). One swab (nerbe plus, Winsen/Luhe, Germany) was used for each teat cup. Four swabs from the same cluster were pooled into one sample. Four milking clusters were used at the farm. One of the clusters was not used on seven occasions for technical reasons. Therefore, only 89 samples (instead of 96, 24 × 4 clusters) were analyzed.

Each sample was covered with 18 mL PW and homogenized as previously described.

During the study, intermediate disinfection of the teat cups with 3% peracetic acid between cows was introduced on the farm. The disinfection was introduced to achieve better milking hygiene between cows due to the poor udder health of some cows. However, we continued to take samples from the teat cup at the end of the completed milking process of all cows.

#### 2.2.4. Milk Filter Samples

The milk filter was installed at the end of the milk pipeline, at the start of the milking process, i.e., all milk collected on that day passed through the filter. One milk filter was taken on each sampling day (*n* = 24). The filters measured 6 cm × 52.5 cm, and the pore size ranged from 100 µm to 250 µm. After the milking process was finished, the milk filter was removed and transferred to a plastic bag. In the laboratory, it was homogenized after being covered with 35 mL PW.

#### 2.2.5. Boot Sock Samples

Socks (romerlabs, surface boot cover swabs, 10001911 (BTSW200BPW)) were placed on shoes before sampling. One pair of boot socks was taken on each sampling day (*n* = 24). The entire barn corridor was walked with the boot socks (approx. 80 steps), avoiding fresh fecal pats. After sampling, the socks were placed in a stomacher bag and transported to the laboratory in a cool box. At the laboratory, 180 mL PW, enough to cover the socks, was added to the stomacher bag, and the same procedure as described above was applied for homogenization.

#### 2.2.6. Fecal Samples

Fecal samples were obtained from the recta of twelve cows, using gloved hands and a lubricant gel. On one sampling day, only eleven cows were sampled. Therefore, 287 samples were analyzed (instead of 288, 12 cows × 24 samplings). The samples were placed in plastic cups with a screw-on lid. Gloves were changed between individual cows to avoid cross-contamination. In the laboratory, 10 g of feces per sample was transferred into stomacher bags and mixed with 90 mL PW. Three scoring categories were used to assess the consistency of the feces (K1: liquid; K2: mushy (normal) consistency; K3: dry and compact). The samples were homogenized individually for 120 s at 4000 rpm.

### 2.3. Microbiological Analysis

#### 2.3.1. Detection and Quantification of Microorganisms

The detection limits of all microorganisms in the samples are provided in Table S1.

*Escherichia coli* (beta-glucoronidase-positive), TACC and *Pseudomonas* spp. were quantified according to ISO 16649-2:2001 (using the spread plate method instead of the pour plate method), ISO 4833:2015 and ISO 13720:2010, respectively. The samples were further diluted 1:10 in PW, and 100 µL per dilution step was spread on agar plates. *Escherichia coli* was cultured on tryptone bile X-glucuronide (TBX) agar (Oxoid Deutschland GmbH, Wesel, Germany) for 24 h at 41.5 °C, TACC on plate count agar (carl roth^®^ GmbH + Co., KG, Karlsruhe, Germany) for 72 h at 30 °C and *Pseudomonas* spp. on cephaloridine fucidin cetrimide (CFC) agar (Merck KGaA, Darmstadt, Germany) for 24 h at 25 °C. Thermophilic *Campylobacter* spp. were detected according to ISO 10272-1:2017, using modified charcoal cefoperazone deoxycholate agar (mCCDA, mixture of Merck & Co., Kenilworth, NJ, USA and Oxoid; 48 h, 41.5 °C). For the enrichment of *Campylobacter* spp., Preston broth (Merck KGaA, Darmstadt, Germany; 24 h, 41.5 °C) was used. The enrichment for teat swabs, milk, swabs from milk clusters and milk filters was performed using 5 mL of the sample dilution and 45 mL of Preston broth. For boot sock samples, 1 mL of sample dilution was used in 9 mL of Preston broth. One gram of fecal sample was weighed into a test tube and covered with 9 mL of Preston broth.

Concurrently, ISO 10272-2:2017 was used for the enumeration of thermophilic *Campylobacter* spp. The samples were further diluted 1:10 in PW, and 100 µL per dilution step was spread on agar plates. In addition, 1 mL of the initial sample (raw milk) or sample suspension (all other sample matrices) was spread on agar plates.

#### 2.3.2. Species Identification

One colony from each sample was selected for *Campylobacter* spp. identification. *Campylobacter* spp. colonies were sub-cultured on Columbia blood agar plates with defibrinated sheep blood (Oxoid Deutschland GmbH, Wesel, Germany) in a microaerobic atmosphere for 24 h at 41.5 °C. Afterwards, further analyses were performed according to ISO 10272-1:2017. In short, characteristic morphology and motility were observed via phase contrast microscopy. A catalase activity test was performed by streaking a loop of culture into a drop of hydrogen peroxide solution on a clean microscope slide. The test was positive if bubbles appeared within 30 s. The detection of cytochrome oxidase activity was performed using a Bactident™ Oxidase test strip (Merck KGaA, Darmstadt, Germany), following the manufacturer’s instructions. A color change to violet/blue indicated that hydrolysis had taken place.

In addition, the genus and species identification of the colonies from sheep blood agar plates was performed using a Bruker MALDI-TOF Biotyper System (Bruker Scientific LLC, Billerica, MA, USA). Colonies were transferred to the MALDI-TOF target and covered with 1.0 µL of α-cyano-4-hydroxycinnamic acid, according to the manufacturer’s instructions (Bruker Scientific LLC, Billerica, MA, USA). The reference database for species identification was provided by Bruker Scientific LLC (MBT-BDAL-8468).

#### 2.3.3. Somatic Cell Count

Somatic cell counts were determined using a simple cell count meter (DCC, DeLaval; Glinde, Germany). Sixty microliters of raw milk from individual cows was loaded into the cassette, and the measurement was carried out according to the operating manual.

### 2.4. Weather Data

Weather data were acquired from an official weather station close to the farm (https://openweathermap.org/ (accessed on 22 June 2022)). The meteorological data collected were temperature (°C) (hourly), pressure (hPa), humidity (%), wind (m/s) and rain (mm/h) data.

### 2.5. Statistical Analysis

Microsoft Excel^®^ 2016 (Microsoft Corp., Redmond, WA, USA) was used to store the data. The software R, version 4.2.1 (Vienna, Austria) [31], was used for data analysis.

#### 2.5.1. Multi-Level Modeling

The effect of the environment data and cross-contamination was evaluated using multi-level modeling, which clusters the observations for each cow (repeated measurements) and offers variation effects on both the sample and cow level.

Due to the large number of zero values in the final results, a multivariate generalized linear regression model was not possible because numerous zero values result in heteroscedasticity and collinearity. They also caused the distribution to be skewed and non-normal. As a result, the *Campylobacter* spp. concentration data were classified as a binary variable (0 and 1), and a multi-level mixed logistic regression was performed. Level one of the model comprised the bi-weekly observations for each cow. Level two consisted of the cows.

Only the teat and feces samples were selected as dependent variables for modeling since the occurrence of *Campylobacter* spp. in almost all other samples was negative. The occurrence of *Campylobacter* spp. in the teats and feces was modeled against weather data (minimum temperature, pressure, wind and humidity), seasons, the concentration values of other microorganisms (*E. coli*, *Pseudomonas* spp. and TACC), teat cleanliness scores, fecal consistency scores and somatic cell count. Each parameter was added to the model in a stepwise manner, and the goodness-of-fit was decided based on the Akaike information criterion (AIC). Due to the relatively small number of observations and the difficulty in merging the model, only parameters with a significant effect were retained in the final model. To test whether the effect of minimum temperature was related to the occurrence of *Campylobacter* spp. in the teat or feces samples, an interaction variable was integrated into the multi-level model.

The intraclass correlation coefficient (ICC) was calculated to evaluate the variation in concentration values between the cows (Level 2) and for each cow throughout the year (Level 1). Multi-level modeling was performed using the lme4 package [32].

#### 2.5.2. Correlation Analysis

The correlation of the concentration of *Campylobacter* spp. on teat skin with its concentration in feces was graphically and statistically assessed using the Spearman rank correlation coefficient for non-parametric data.

## 3. Results

### 3.1. Species Identification

*Campylobacter* spp. were isolated from 263 of the 997 samples tested. Of the 263 isolates, 256 (97.3%) were identified as *C. jejuni* and 7 as *C. hyointestinalis* (2.7%). The latter was only isolated from feces. Five of the seven isolates were obtained from one cow (6001). The remaining two isolates were obtained from two other cows (4664 and 4652). All *Campylobacter* spp. isolates were positive for catalase activity and cytochrome oxidase activity.

### 3.2. Prevalence and Concentration Data

An overview of all prevalence data for the specific sample types and analyzed bacteria is shown in Table 1.

The prevalence of *Campylobacter* spp. was the highest in fecal samples (77.1%), followed by boot sock samples (29.2%), teat swabs (12.2%), milk filters (4.2%) and raw milk samples (0.4%). No *Campylobacter* spp. were detected in the milking clusters.

*Escherichia coli* was most frequently detected in the fecal samples (94.8%), boot sock samples (100%) and teat swab samples (81.8%). *Pseudomonas* spp. were most frequently detected on the teats (97.5%), in the milk filters (95.8%), the milking clusters (51.7%) and in raw milk (71.7%). Fecal samples were not tested for *Pseudomonas* spp.

The somatic cell count in the 286 milk samples ranged from 3 to 933 × 10^3^ cells/mL. One sample taken on 7 July 2020 had a somatic cell count of 4.7 × 10^6^ cells/mL. This sample appeared normal, without signs indicative of inflammation such as flocculation.

An overview of the quantitative data on all bacterial microorganisms in all sample types can be found in Table 2.

*Campylobacter* spp. were only detected in one raw milk sample and one milk filter, with a concentration of 2.37 log_10_ CFU/mL and 2.74 log_10_ CFU/filter, respectively. These samples were taken on the same sampling day. Otherwise, the highest mean concentration of *Campylobacter* spp. detected in the boot sock samples was 3.01 ± 1.05 log_10_ CFU/2 socks and 2.43 ± 0.9 log_10_ CFU/g in the cow feces. The mean concentration of *Campylobacter* spp. at the cow teats was 1.26 ± 0.75 log_10_ CFU/4 teats. It is important to note the different units in the concentration data.

### 3.3. Campylobacter *spp.* Prevalence and Concentration in Feces

**Figure 1:**
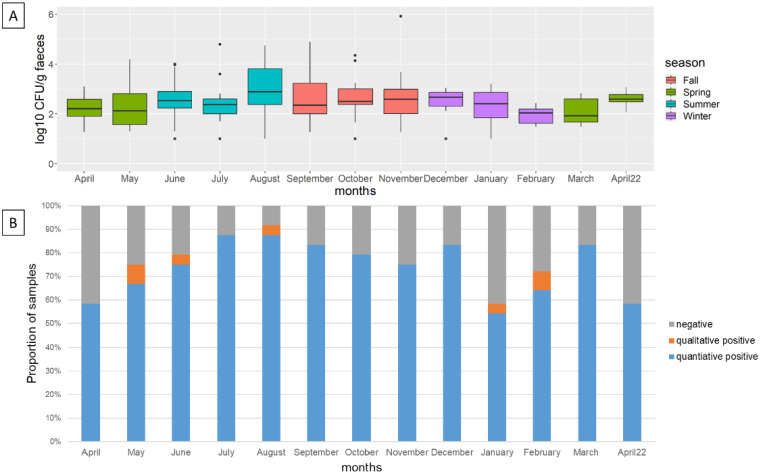
(**A**) Concentration of *Campylobacter* spp. in feces per month. (**B**) Proportion of negative, qualitative positive (enrichment) and quantitative positive samples.

The fecal consistency scores were not related to the *Campylobacter* spp.-positive fecal samples. In total, 26, 191 and 70 teat swab samples were categorized as K1, K2 and K3, respectively. The scoring for the positive *Campylobacter* spp. samples ranged from K1 to K3 (Table S2). A seasonal overview indicated that *Campylobacter* spp. could be detected in the herd’s feces throughout the year. The mean concentration of the positive *Campylobacter* spp. samples ranged between 1.9 log_10_ CFU/g and 2.9 log_10_ CFU/g (Figure 1A). The proportion of negative samples was lower in the warm months (July and August) compared to colder months (November, January, February and April) except for December and March (Figure 1B).

**Figure 2:**
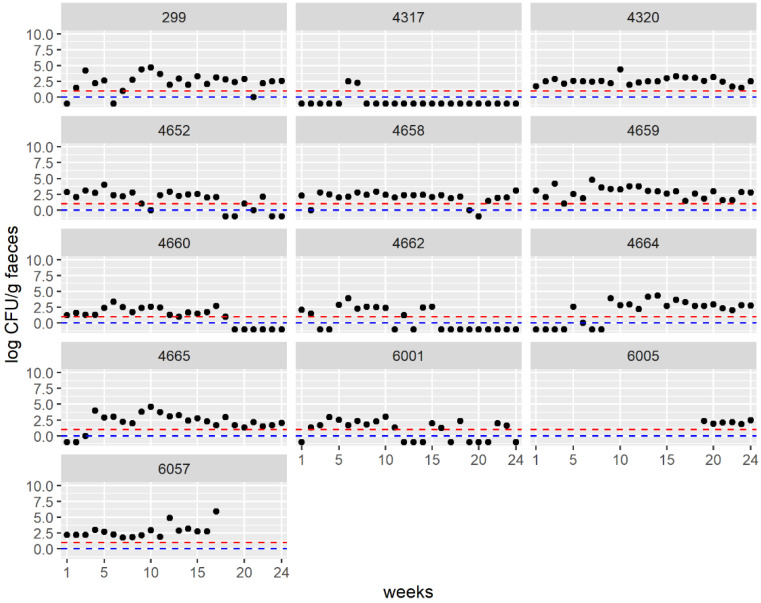
Overview of the concentration of *Campylobacter* spp. in feces of individual cows (ID numbers). In total, 24 samplings were performed over one year. The first sampling took place in April (week 1). Cow 6057 was replaced by cow 6005 after the 17th sampling. The limits of quantification and detection for enrichment are depicted as a red and blue line, respectively. Dots below the limit of detection indicate negative samples. Dots at the blue line indicate samples that were qualitatively positive but could not be quantified.

The concentration of *Campylobacter* spp. in the feces of individual cows over time is depicted in Figure 2 and Table S2. Occasionally, no *Campylobacter* spp. were detected in individual cows. All cows carried *Campylobacter* spp. in at least two samples. Cow 4317 only tested positive for *Campylobacter* spp. on two consecutive sampling occasions in July. Cows 4320, 4659 and 6005 were always positive.

The highest median concentrations in the feces of individual cows shedding *Campylobacter* spp. were 2.9 ± 0.9 log_10_ CFU/g (4659), 2.8 ± 0.96 log_10_ CFU/g (4664) and 2.7 ± 1.09 log_10_ CFU/g (6057) and the lowest were 1.6 ± 0.68 log_10_ CFU/g (4660) and 2.0 ± 0.57 log_10_ CFU/g (6001).

### 3.4. Campylobacter *spp.* in Teat Swab Samples

Among the 286 teat swab samples, 35 were positive for *Campylobacter* spp. These originated from 15 different cows (Table S3). Teat swab samples from the individual cows were positive for *Campylobacter* spp. on up to three occasions. On one sampling day (14 June 2021), nine teat swab samples were positive for *Campylobacter* spp. On the same day, the positive raw milk sample and the positive milk filter were obtained. The positive raw milk sample was from one cow (6005) tested as a replacement for another cow in the dry period. Therefore, no fecal sample was collected from this cow.

On the other sampling days, a maximum of two teat swab samples were positive for *Campylobacter* spp. The cleanliness scores of the teat samples could not be linked to the *Campylobacter* spp. positive teat swab samples. In total, 23, 117, 93 and 53 teat swab samples were categorized as K1, K2, K3 and K4 respectively. The scoring for the positive *Campylobacter* spp. samples ranged from K1 to K4 (Table S3).

**Figure 3:**
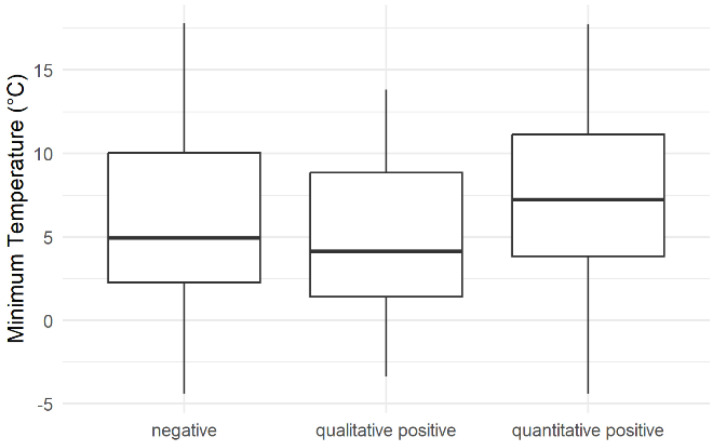
Association of minimum temperature with the occurrence of *Campylobacter* spp. on teat skin (*Campylobacter* spp. negative, qualitatively positive (enrichment) and quantitatively positive teat swab samples). The black line indicates the median temperature, and the boxes display the interquartile range.

*Campylobacter* spp. positive teat swab samples were detected at minimum outdoor temperatures between −4 °C and 17 °C (Figure 3). The negative samples were observed at all minimum temperatures. The qualitative positive samples occurred within a smaller temperature range. The mean minimum temperature for the detection of negative, qualitative and quantitative positive samples were 5 °C, 4 °C and 7 °C, respectively.

### 3.5. Multi-Level Model and Correlation Analysis

The multi-level model (Table 3) with a binary outcome of *Campylobacter* spp. (dependent variable) shows the effective parameters on the occurrence of *Campylobacter* spp. in the teat swab and fecal samples.

The seasons fall, spring and summer were individually compared to the winter. The effect of the season as a whole also significantly improved the model fit. The effect of the sample type is shown by the comparison of the teat and feces samples.

An integrated interaction term between the minimum temperature and the sample type (feces or teat swab) demonstrated a different temperature effect of the occurrence of *Campylobacter* spp. for the two sample types.

Other parameters tested, including the weather conditions (humidity, wind and rain), other microorganisms (*Pseudomonas* spp. and TACC), the scoring of teat and fecal samples and somatic cell counts, did not show an influence on the occurrence of *Campylobacter* spp.

The ICC of the multi-level model was 0.11, indicating that 11% of the variation in the model comes from the variation between the cows, whereas the rest of the heterogeneity originates from the variation in the measurements for each cow that happened throughout the year.

Correlation analyses were performed for the concentrations of *Campylobacter* spp. in teat swab samples and the concentrations of *Campylobacter* spp. in the feces samples. No correlations were detected in these analyses.

## 4. Discussion

To explore the risk associated with the consumption of raw milk, the occurrence of *Campylobacter* spp. in a small German dairy herd was studied. In our study, the highest proportion of positive samples was found in the feces (77.1%), and the lowest proportion of positive samples was found in the milking clusters (0%) and raw milk (0.4%, one sample).

The high prevalence of *Campylobacter* spp. in the cows’ fecal samples is consistent with some previous studies, which reported a prevalence between 66.7% and 78.5% [21,27,30,33]. A lower prevalence (7–38%) of *Campylobacter* spp. in cow feces was reported in other studies [20,26,34,35]. Two of these studies used comparable study designs and detection methods [26,34]. One study did not mention the interval between sampling and testing [20]. The last study reported that the samples were analyzed one day after sampling [35]. The interval between sampling and testing could have influenced the detection of *Campylobacter* spp. in the samples as *Campylobacter* spp. is a fastidious organism (with a low oxygen tolerance and sensitivity to temperature and pH) [36]. In addition, a low prevalence was observed in a study carried out on smaller farms, while a larger farm displayed a higher prevalence [26].

This is the first study to monitor the concentration of *Campylobacter* spp. in individual dairy cows in Germany over a period of one year using rectal fecal sampling. The concentration of *Campylobacter* spp. in the fecal samples varied between individual cows. It ranged between high concentrations (over 5 log_10_ CFU/g) and negative samples. Some cows shed *Campylobacter* spp. consistently (4320, 4659), whereas other cows shed *Campylobacter* spp. less often (4662) or only twice (4317) throughout the year. The proportion of negative samples was lower in the warmer months than in colder months, whereas the mean concentration in any individual month was relatively constant, between 1.9 log_10_ CFU/g and 2.9 log_10_ CFU/g.

The overall mean concentration of *Campylobacter* spp. in fecal samples determined in this study was 2.43 ± 0.9 log_10_ CFU/g. A similar value of 2.1 ± 0.45 log_10_ CFU/g was reported in a Danish study [20]. A longitudinal study in New Zealand examined the concentration of *Campylobacter* spp. in feces from individual cows on pasture and in confinement housing [23]. The concentration of *C. jejuni* varied between 0 and 6.0 log_10_ CFU/g in the herd on pasture and between 0 and 5.7 log_10_ CFU/g in the confinement-housed herd. The median concentration of *Campylobacter* spp. in feces per cow was between 2.9 log_10_ CFU/g and 1.6 log_10_ CFU/g for the individual cows. Significant differences in the frequency and range of the *C. jejuni* concentrations occurred among individual cows. At least three cows in the two different herds were identified as high shedders of *Campylobacter* spp., with a median concentration between 3.3 log_10_ to 3.6 log_10_ CFU/g [23]. Our study underscores the previous finding that cows excrete *Campylobacter* spp. intermittently [14,23,26], although according to the criteria of the New Zealand study, the cows we sampled would not be classified as high shedders.

Overall, 29.2% of the boot sock samples tested positive for *Campylobacter* spp., with a mean concentration in positive samples of 3.01 ± 1.05 log_10_ CFU/2 socks. The samples were taken in the barn corridor, avoiding direct contact with fresh cowpats. The positive boot sock samples were found once each in January, May, June and July. In November, both boot sock samples were positive. Both the prevalence and the concentration values show that *Campylobacter* spp. can be found in the barn environment, and that this can represent a contamination risk throughout the year.

Another study found a higher prevalence (60%) of *Campylobacter* spp.-positive boot sock samples [27]. However, they did not avoid fresh dung pats, and it was described that all parts of the socks were in contact with the feces [27]. The intensity of the fecal contamination of the boot sock samples could explain the differences in the concentrations found in our study.

The finding of *Campylobacter* spp. in 12.2% of teat swabs with a mean concentration of 1.26 ± 0.75 log_10_ CFU/4 teats underlines that the teat skin can become contaminated with *Campylobacter* spp. The origin of these bacteria is likely the fecal contamination of the environment. The occurrence of *Campylobacter* spp. in teat swab samples was not at a specific minimum temperature (Figure 3), although the multi-level model indicated the minimum temperature as an effective parameter. However, the weak but reliable effect of the temperature on the occurrence of *Campylobacter* spp. was based on feces and teat swab samples. An interaction term indicated that both sample types were influenced differently. However, the number of positive teat swab samples is too small to effectively analyze the effect. Further, the negative, qualitative positive and quantitative positive samples all lay in the same temperature range. The minimum temperature was used for the analysis since the teat swab samples were taken early in the morning. Previous *in vitro* studies demonstrated a slower inactivation of *C. jejuni* by oxygen at cooler temperatures [37,38,39]. This was not observed in our study, as all classes of results occurred in the same temperature range.

In this study, the raw milk samples were taken after the teats had been cleaned. No sterile milk sampling with teat disinfection was performed to mimic the routine milking situation. Only one raw milk sample (0.4%) was positive. The *Campylobacter* spp. concentration in the raw milk sample was 2.37 log_10_ CFU/mL. The contamination of the milk sample indicates that not all *Campylobacter* spp. had been removed from the teat skin by the routine cleaning process. A concentration of *Campylobacter* spp. of 2.74 log_10_ CFU/filter was detected in the milk filter on the same sampling day. The entry of *Campylobacter* spp. into the milk pipeline, as indicated by the positive milk filter, could have occurred through the transmission of *Campylobacter* spp. from the teat skin to the milk during milking, which is in line with the positive milk sample on the same day. In addition, the nine positive teat swab samples on the same sampling day indicated a cross-contamination event of the raw milk and milk filter samples. The milking clusters did not test positive for *Campylobacter* spp. and were therefore not assumed to be an entry source. A recent meta-analysis estimated the prevalence of *Campylobacter* spp. in raw milk samples at 1.18% [13]. Two studies estimated concentration data for *Campylobacter* spp. in raw milk using the MPN method [14,15]. They found low concentrations of ≤5 MPN/100 mL, but one outlier of 100 MPN/100 mL was detected [14]. In the other study, the *Campylobacter* spp. level of one sample was 1 CFU/21 mL of raw milk from the farm vats [15].

A recent study indicated that there is only a limited detection of *Campylobacter* spp. CFU in raw milk, possibly due to the *Campylobacter* spp. entering a viable but non-culturable (VBNC) state [40]. This was underlined by a newly developed viable qPCR using propidium monazide (PMA). This qPCR allows for the detection of intact and putatively infectious units (IPIUs) comprising CFU and VBNC bacteria. It demonstrated an underestimation of the survival of *Campylobacter* spp. with a difference of up to 4.5 log_10_ between the CFUs and IPIUs. Furthermore, within a certain time period, the CFUs of those IPIUs could be restored using a special “low-oxygen” atmosphere, confirming the viability of the bacteria [40]. However, in field samples, the application of the viable qPCR method is difficult due to the low concentration of *Campylobacter* spp. in raw milk, and the detection limit and application of different atmospheres is beyond the current ISO 10272-1.

Recent studies have shown that milk filter sampling is a potential approach to assessing the risk of *Campylobacter* spp. contamination in milk. The milk filter is installed at the end of the milking line so that all raw milk from all cows passes through it before ending up in the bulk tank. The reported prevalence of positive milk filters ranged from 0–14% [27,28,30,41]. In some studies, none of the concurrently collected bulk milk tank samples were positive, or a low prevalence was detected. In our study, one milk filter was positive, with a concentration of 2.74 log_10_ CFU/filter. On the same sampling day, nine teat swab samples and one raw milk sample were positive.

In summary, our data indicate a low risk of *Campylobacter* spp. contamination in the raw milk of the herd under study. Still, even in such a small herd, the contamination of milk can occur sporadically. Further research is required to better understand the reasons for sporadic contamination events.

We have used a multi-level logistic model to investigate the effective parameters on the presence of *Campylobacter* spp. in the feces and teat swab samples. The ICC shows that 11% of the variation in the occurrence was between the cows, whereas the rest of the variation happened throughout the year for each cow. The effect of the seasons was confirmed in the multi-level model, indicating the significant difference between the summer and winter. Based on our model, temperature affects the concentration of *Campylobacter* spp. in teat and feces samples differently. However, other weather data were not shown to have an effect.

We used *E. coli* as a fecal contamination indicator since feces are considered the primary source of milk contamination during or after the milking process [42,43]. *Pseudomonas* spp. were used as an indicator of environmental contamination. We could show that the prevalence of *E. coli* was the highest in the feces, on the teat swab and in the boot sock samples. In contrast, the prevalence of *Pseudomonas* spp. was the highest on the teat swab and in the milk filter, milk equipment and raw milk samples. The *Escherichia coli* concentration data comprised a parameter effecting the occurrence of *Campylobacter* spp. in the feces and teat swab samples. This strengthens the assumption that the cross-contamination of teats with *Campylobacter* spp. had a fecal origin. However, *Pseudomonas* spp. and TACC had no effect on the occurrence. Another study assumed a relationship between the presence of *C. jejuni* in the bulk tank milk and a high load of *Enterobacteriaceae* in the same samples [6]. They found no association and supposed that fecal contamination might not be the only mechanism responsible for the presence of *C. jejuni* in raw milk. It has been suggested that udder infection may play a role in raw milk contamination, whereby the pathogen is directly excreted into the raw milk [6,44,45]. Unfortunately, the somatic cell count was not measured in any of these studies.

The health status of the studied cows is often not reported [18]. *Campylobacter* spp. commonly colonizes the intestine of asymptomatic cows [19,25,35,46,47,48,49,50]. Our data confirm that *Campylobacter* spp. are part of the intestinal microbiota of healthy dairy cattle.

In one sample, 7 July 2022, there was a somatic cell count of 4.7 × 10^6^ cells/mL. In this sampling event, we found no association between the occurrence of *Campylobacter* spp. and the high somatic cell count. In the following sampling runs, this cow was excluded from the milk samples for as long as the local antibiotic treatment continued. The *Campylobacter* spp.-positive raw milk sample in this study had a low somatic cell count of 11 × 10^3^ cells/mL. However, there was only one positive sample, and future research might therefore be necessary to determine whether there is an association of *Campylobacter* spp. with high somatic cell counts.

The scoring was used to either monitor whether the cows had diluted feces or a severe fecal contamination of the teats. In the experimental setup of this study, the teat scores were not found to be a parameter that influenced the occurrence *Campylobacter* spp. in the multi-level model. Another study also found no association between scores and the detection of *Campylobacter* spp. in bulk milk tanks, milk filters or feces. They demonstrated an association between cow hygiene and the detection of *Campylobacter* spp. in the teat milk [30]. However, they used a mean score calculated for the herd at each visit and not only a teat skin score directly related to the *Campylobacter* spp. concentration.

This study demonstrated that the contamination of raw milk with *Campylobacter* spp. was a rare event, although the cows were consistently colonized with *Campylobacter* spp. in the intestine, and cross-contamination of the teats with *Campylobacter* spp. did occur. On one sampling day, nine teat swab samples, one raw milk sample and the milk filter tested positive for *Campylobacter* spp. In terms of the annual study, we could not demonstrate a parameter that influenced this sporadic contamination event.

## 5. Conclusions

The obtained data can be integrated into risk assessments for *Campylobacter* spp. along the raw milk supply chain. The occurrence of *Campylobacter* spp. in feces differs between individual cows throughout the year. The season, *E. coli* concentration, minimum temperature and sampling type are effective parameters for the occurrence of *Campylobacter* spp. in feces and teat swab samples. No correlation was observed between the concentrations of *Campylobacter* spp. in feces and teat swab samples. Further research is required to explain sporadic *Campylobacter* spp. contamination in raw milk.

## Figures and Tables

**Table 1:** Prevalence data for all sample types and taxa analyzed.

Sample Type	Total Number of Samples	Proportion of Positive Samples (%)		
		*Campylobacter* spp.	*E. coli*	*Pseudomonas* spp.
Teat swab	286	12.2	81.8	97.6
Raw milk	286	0.4	15.0	71.7
Milking cluster	89	0	15.7	51.7
Milk filter	24	4.2	45.8	95.8
Feces	287	77.1	94.8	Not tested
Boot socks	24	29.2	100	58.3

**Table 2:** Mean log_10_ concentration and standard deviation for all sample types and microorganisms analyzed.

Sample Type [Unit]	Concentration Data (No. of Positive Samples)			
	*Campylobacter* spp.	*E. coli*	*Pseudomonas* spp.	TACC ^1^
Teat swab [log CFU/4 teats]	1.26 ± 0.75 (35)	3.87 ± 0.98 (234)	8.03 ± 0.62 (279)	5.36 ± 0.71 (284)
Raw milk [log CFU/mL]	2.37 (1) ^2^	2.47 ± 0.53 (43)	2.7 ± 0.6 (205)	4.96 ± 0.66 (286)
Milking cluster [log CFU/4 cups]	0 (0)	2.69 ± 0.55 (14)	2.78 ± 0.46 (46)	5.07 ± 0.66 (84)
Milk filter [log CFU/filter]	2.74 (1) ^2^	3.74 ± 0.76 (11)	5.16 ± 0.9 (23)	6.91 ± 0.54 (24)
Feces [log CFU/g]	2.43 ± 0.9 (215)	4.48 ± 1.18 (273)	Not tested	6.34 ± 0.48 (285)
Boot socks [log CFU/2 socks]	3.01 ± 1.05 (4)	5.39 ± 1.11 (24)	6.64 ± 0.52 (14)	9.18 ± 0.63 (23)

^1^ total aerobic colony count; ^2^ no standard deviation calculation possible.

**Table 3:** Multi-level model with parameters with an effect on the occurrence of *Campylobacter* spp. in teat swab and feces samples. The confidence intervals (CI) demonstrate the variability of the odds ratio (OR). When confidence interval contains 1, the effect is not significant.

	Effect Estimate (OR)	Lower CI	Upper CI
Fall	2.88	0.82	10.676
Spring	0.72	0.23	2.25
Summer	15.02	2.15	121.189
Teats	0.01	0.00	0.03
Log_10_ *E. coli*	1.56	1.2	2.11
Minimum temperature	0.98	0.84	1.13
Type Minimum Temperature (interaction term)	0.79	0.67	0.92

## Data Availability

Data is contained within the article or Supplementary Material.

## Supplementary Materials

The following supporting information can be downloaded at: https://www.mdpi.com/article/10.3390/foods12081639/s1, Table S1: Detection limits of microorganisms. Table S2: Concentration data for *Campylobacter* spp. in feces of individual cows per sampling. Table S3: Concentration data for *Campylobacter* spp. on teat swab samples of individual cows per sampling.
